# The Renoprotective Actions of Peroxisome Proliferator-Activated Receptors Agonists in Diabetes

**DOI:** 10.1155/2012/456529

**Published:** 2012-02-12

**Authors:** M. C. Thomas, K. A. Jandeleit-Dahm, C. Tikellis

**Affiliations:** Division of Diabetic Complications, Baker IDI Heart and Diabetes Institute, P.O. Box 6492, Melbourne, VIC 8008, Australia

## Abstract

Pharmaceutical agonists of peroxisome proliferator-activated receptors (PPARs) are widely used in the management of type 2 diabetes, chiefly as lipid-lowering agents and oral hypoglycaemic agents. Although most of the focus has been placed on their cardiovascular effects, both positive and negative, these agents also have significant renoprotective actions in the diabetic kidney. Over and above action on metabolic control and effects on blood pressure, PPAR agonists also appear to have independent effects on a number of critical pathways that are implicated in the development and progression of diabetic kidney disease, including oxidative stress, inflammation, hypertrophy, and podocyte function. This review will examine these direct and indirect actions of PPAR agonists in the diabetic kidney and explore recent findings of clinical trials of PPAR agonists in patients with diabetes.

## 1. Introduction

Pharmaceutical agonists of peroxisome proliferator-activated receptors (PPARs) are widely used in the management of type 2 diabetes. PPAR*α* agonists, known as fibrates, have been used for over 40 years in patients with diabetes, chiefly as lipid-lowering agents. Over the last decade, PPAR*γ* agonists, known as thiazolidinediones (TZDs) or glitazones, have also come into clinical use as oral hypoglycaemic agents. Selective agonists of a third isoform of PPAR, PPAR *β*/*δ* are also under clinical development for treatment of the metabolic syndrome [1]. Although most focus has been placed on their metabolic and cardiovascular effects, these agents also have direct and indirect actions in the diabetic kidney. Such actions are potentially important as the presence and severity of kidney disease adversely affects the well being of individuals with diabetes and significantly contributes to disease morbidity and increases their risk of a premature death. For example, we have shown that in Finnish adults with type 1 diabetes excess mortality associated with diabetes is almost entirely confined to those with chronic kidney disease (CKD) [2]. Equally, in patients with type 2 diabetes, kidney disease is associated with an increase in the risk of death [3, 4]. Consequently, long-term benefits from preventing and managing diabetic kidney disease may prove to be among the most important actions of these agents. This review will examine the indirect and direct actions of PPAR agonists specifically in the diabetic kidney and explore recent findings of clinical trials of PPAR agonists in patients with diabetes.

## 2. The Expression of PPARs in the Kidney

PPARs are ligand-activated nuclear transcription factors that have complex biologic effects, resulting from the transactivation or transrepression of dozens of genes [5]. Transactivation effects require dimerisation of PPAR with retinoid X receptor (RXR), followed by translocation to the nucleus where upon the PPAR: RXR dimer binds to the PPAR response element of target genes and induces the expression of these genes [5]. Ligand-dependent transrepression is mediated via interference with nuclear receptors such as activator protein-1 (AP-1) and nuclear factor-*κ*B (NF-*κ*B) [6]. The relative importance of activation versus repression pathways for the renal action of PAAR agonists remains to be established. Moreover, there is evidence that not all PPAR ligands stimulate transactivation and transrepression pathways to a similar extent [7], and their relative importance may be different in different tissues.

In the kidney, PPAR*α* is expressed in proximal tubules and medullary thick ascending limbs where it is thought to be involved in the regulation of protein-degradation systems through maintenance of ATP homeostasis [8], control of fatty acid *β*-oxidation [9], and regulation of cytochrome P450 in proximal tubules [10]. PPAR*γ* is predominantly expressed in medullary collecting ducts and pelvic urothelium [11–13], the latter site is potentially important for the putative link between PPAR*γ* agonists and transitional cell cancer [14]. Studies using more specific antibodies suggest that lower level PPAR*γ* expression is observed in glomeruli, proximal and distal tubules, the loop of Henle, medullary collecting ducts, and intima-media of renal vasculature [15]. The third isoform of PPAR, PPAR *β*/*δ* is also ubiquitously expressed in the kidney, with the highest levels observed in the proximal straight tubule in renal cortex and medulla [16].

The expression and activity of PPARs is significantly modified by diabetes, partly reflecting the abnormal metabolic milieu and partly contributing to it. For example, the expression of PPAR*α* is markedly reduced in pancreatic islets of obese prediabetic Zucker diabetic fatty rats [17] and in isolated rat pancreatic islets in response to elevated glucose levels [18]. However, the expression of PPARs in the diabetic kidney appears to be generally increased. For example, the expression of PPAR*α* is upregulated in glomeruli and cortical tubules of diabetic db/db mice [19] and in renal cells following exposure to high glucose levels [20]. Similar findings have also been reported with the induction of PPAR*γ* [21, 22] and PPAR *β*/*δ* [23] expression in the diabetic kidney. Increased expression of PPAR*α* and PPAR*γ* has also been described in renal biopsies from patients with CKD, correlating inversely with extent of proteinuria and kidney function [24].

## 3. The Renoprotective Actions of PPAR***α*** Agonists

### 3.1. Experimental Studies

**Figure 1:**
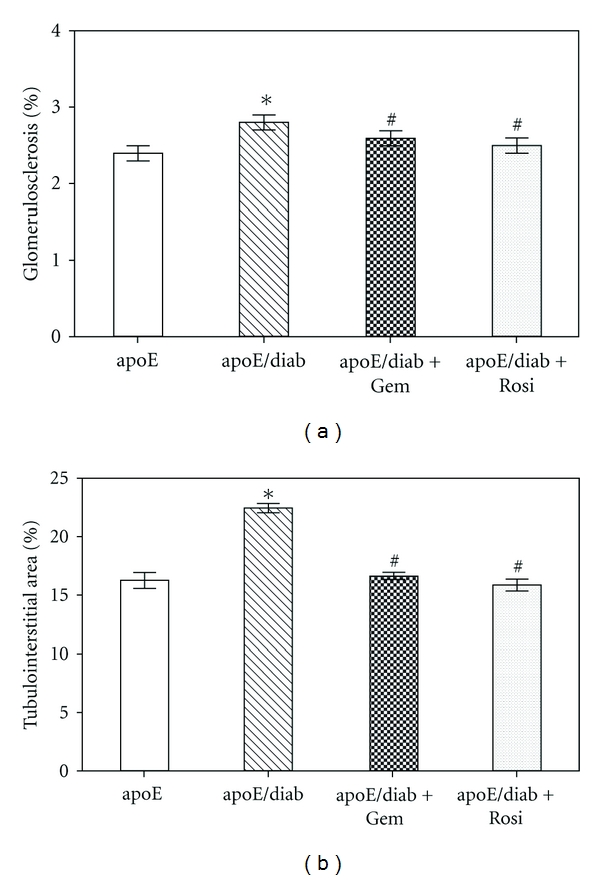
Treatment with PPAR agonists, gemfibrozil and rosiglitazone reduces glomerulosclerosis (a) and tubulointerstitial expansion (b) in streptozotocin diabetic apolipoprotein E knockout mice [25]. Data shows mean ± SEM; *versus control *P* < 0.05, ^#^versus diabetes *P* < 0.05).

There is strong evidence that PPAR*α* agonists both have independent renoprotective actions in experimental diabetes. For example, we have shown that treatment with the PPAR*α* agonist, gemfibrozil (100 mg/kg/day), is able to attenuate albuminuria, glomerulosclerosis, tubulointerstitial expansion, and collagen IV deposition associated with streptozotocin-induced diabetes (Figure 1) [25]. Importantly, this is a model of type 1 diabetes, meaning that these renoprotective effects are observed in the absence of changes in glucose, insulin, or lipid levels or a reduction in blood pressure, suggesting a direct mechanism of action (see below). Similar renoprotective actions on diabetic kidney disease have also been observed in other rodent models, including *db*/*db* mice, obese Zucker rats and OLETF (Otsuka Long-Evans Tokushima Fatty) rats, although indirect actions through amelioration of dyslipidemia may have contributed to some of these findings [26–31]. By contrast, in diabetic PPAR*α*-knockout mice, diabetic nephropathy is more severe than in wild-type mice [20]. 

### 3.2. Clinical Studies with PPAR*α*

In clinical studies, significant reductions in albuminuria have also been reported following the use of fibrates in patients with diabetes. For example, in the diabetes atherosclerosis interventional study (DAIS) fenofibrate reduced albuminuria and the risk for progression, independent of changes in lipid parameters [34]. Albuminuria was also reduced in the FIELD (fenofibrate intervention and event lowering in diabetes) study in patients with type 2 diabetes, with reduced risk of progression in patients receiving fenofibrate (9.5% versus 11.0%, *P* < 0.05) [35]. Furthermore, regression of albuminuria was also increased in patients receiving fenofibrate (9.4% versus 8.2%, *P* < 0.05). Microalbuminuria was also modestly reduced in the ACCORD-LIPID trial [36]. These renoprotective effects appeared to be independent of the degree of lipid-lowering or starting lipid concentrations, leading to the suggestion that direct effects arising from *PPAR*α** receptor activation may be mediating its benefits. There are very few large studies with other fibrates that included renal function as a secondary outcome, although a few small studies have reported reduction in microalbuminuria with gemfibrozil [37].

### 3.3. Mechanisms of Action

Diabetic dyslipidaemia is a major reversible risk factor for diabetic CKD [38–41]. A range of quantitative and qualitative lipid and lipoprotein abnormalities are observed in patients with diabetes and nephropathy, including increased plasma triglycerides, small, dense LDL, and reduced HDL cholesterol levels [42]. Increased triglyceride levels are largely due to the accumulation of very low density lipoprotein (VLDL), chylomicron remnants, and intermediate density lipoprotein (IDL) particles in the plasma. This is thought to reflect both the overproduction of triglyceride-rich VLDL (due to increased flux of free fatty acids and hepatic resistance to the effects insulin), together with reduced catabolism (associated with reduced of lipoprotein lipase activity) [43]. Although LDL cholesterol levels in patients with type 2 diabetes are often within the normal range, there remain significant disturbances in LDL metabolism in diabetes, especially in patients with nephropathy. For example, LDL production is generally reduced, while impaired turnover of LDL particles [44] promotes glycoxidative modification of lipoprotein particles. These high levels of oxidized LDL increase the production of cytokines associated with inflammation and chemotaxis [41]. Diabetes is also associated with the accumulation of small dense, triglyceride-rich, LDL particles. HDL cholesterol levels are invariably reduced in patients with type 2 diabetes, reflecting increased catabolism of HDL particles. In addition, HDL particles become enriched with triglycerides, in an attempt to cope with an increased VLDL burden.

Activation of PPAR*α* with the use of fibrates in patients with diabetes increases HDL cholesterol [45, 46], decreases triglyceride levels, and shifts LDL-C distribution toward larger particles [47]. On their own, these improvements in the lipid profile would be anticipated to lead indirectly to a reduction in renal dysfunction associated with diabetes. For example, in a high fat feeding model, which specifically results in renal lipotoxicity, PPAR*α* agonists such as fenofibrate can inhibit the development of renal injury [48].

Over and above their effects on lipid metabolism, PPAR*α* agonists also appear to have direct effects on a number of critical pathways that are implicated in the development and progression of diabetic kidney disease. None of these actions occurs in isolation but appear synergistically intertwined, consistent with the key role of the PPAR signalling pathway as a metabolic and cellular regulator. Such actions may not even be mediated through PPAR receptors. For example, there are some data to suggest that PPAR*α* agonists also have activities through PPAR-independent pathways. It is known, for example, that some of the actions of fibrates persist in PPAR*α*-deficient mice [49]. PPAR*α* agonists are also able to directly antagonize the activities of other transcription factors including AP-1, signal transducers and activators of transcription 1 (STAT-1), and NF*κ*B, possibly by transrepression of other nuclear receptors [50, 51].

#### 3.3.1. PPAR*α* Agonists as Renal Antioxidants

**Figure 2:**
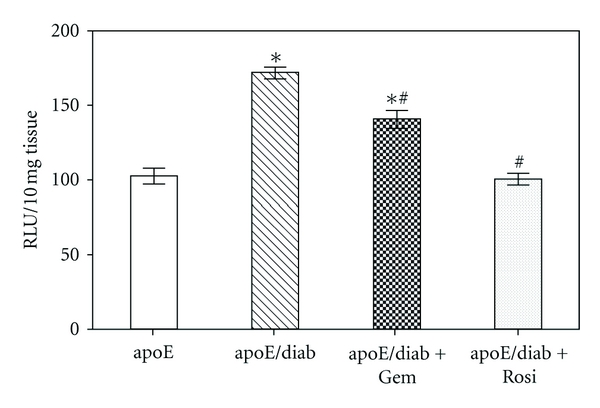
Increased superoxide production in the diabetic vasculature is significantly reduced following treatment with PPAR agonists, gemfibrozil, and rosiglitazone in streptozotocin diabetic apolipoprotein E knockout mice [32, 33].

Renal disease in diabetic patients is characterized by oxidative modification of proteins, lipids, carbohydrates and DNA associated with increased production of oxidants or reactive oxygen species (ROS) that exceeds local antioxidant capacity. In particular, diabetes is associated with the activation of enzymes that directly liberate ROS, including NAD(P)H oxidase [52]. The potential importance of this pathway is illustrated by findings demonstrating that pharmacological inhibition of NAD(P)H oxidase with apocynin prevents mesangial matrix expansion seen in experimental diabetic nephropathy [53]. We have previously shown that NADPH-dependent superoxide production was increased in the vasculature of diabetic mice and that this may be attenuated following treatment with the PPAR*α* agonist, gemfibrozil (Figure 2) [52]. In addition, gemfibrozil attenuated gene expression of each of NAD(P)H oxidase subunits including *Cybb*, *Ncf1* and *Rac1*, the genes encoding gp91phox, p47phox, and Rac-1, respectively. TGF-*β*-induced oxidative stress in mesangial cells can also be attenuated by PPAR*α* agonist, clofibrate [54]. These findings are consistent with those of Evans et al., who demonstrated a reduction in oxidative stress after 3 months of treatment with ciprofibrate in subjects with type 2 diabetes [55]. Another major source and target of ROS in diabetes appears to be the mitochondria [56, 57], which generate the damaging superoxide anion O_2_^∙−^ associated with dysfunction of the mitochondrial respiratory chain. Selective mitochondrial antioxidants, like mitoQ, have been shown to have renoprotective actions in experimental diabetes [58]. Recent data suggest that mitochondrial function may also be modified by PPAR*α* agonists, potentially contributing to their antioxidant and renoprotective actions [59].

#### 3.3.2. PPAR*α* Agonists as Anti-Inflammatory Agents

Inflammation also plays a significant role in the development and progression of diabetic kidney disease, through the action of inflammatory cytokines, leucocyte recruitment, and endothelial dysfunction. Anti-inflammatory strategies have long been known to be effective in diabetic kidney disease. For example, NSAIDs and COX-2 inhibitors both reduce albuminuria in patients with diabetic kidney disease, although these actions are partly haemodynamic due to inhibition of prostaglandin synthesis. PPAR*α* agonists exert a range of anti-inflammatory actions which may be beneficial in diabetic kidney disease. For example, treatment with fibrates is able to suppress circulating levels of cytokines in patients with diabetes [60, 61]. Treatment with fibrates has been shown to reduce renal inflammation and tubulointerstitial fibrosis in diabetic fatty rats, an animal model of type 2 diabetes [62]. In particular, PPAR*α* agonists appear to reduce the expression of key adhesion molecules reducing VCAM expression [63] thereby reducing monocyte recruitment and subsequent activation [64, 65].

#### 3.3.3. PPAR*α* Agonists and Podocyte Biology

The glomerular podocyte plays a key role in the development and progression of albuminuria and glomerulosclerosis associated with diabetes [66–69]. Indeed, recent studies show that mice with specific deletion of the insulin receptor only from their podocytes develop significant albuminuria together with histological features that recapitulate diabetic nephropathy, but in a normoglycemic environment [70]. Such data place podocytes, and more particularly the dysregulation of their growth and differentiation, at the very centre of the pathogenesis of nephropathy. Podocytes clearly express PPAR*α* but more importantly podocyte injury may be attenuated following treatment with PPAR agonists, both *in vitro* [71] and in vivo [27, 72–74]. For example, PPAR*α* agonists are also able to increase gene expression of the key slit-pore protein, nephrin, in diabetic nephropathy, potentially contributing to their antiproteinuric actions [27].

### 3.4. Other Renal Actions of PPAR*α* Agonists

The renal action of PPAR agonists are made more complicated by their actions on creatinine clearance and sodium retention, attributable to PPAR*α* and PPAR*γ* activation, respectively. Treatment with fibrates is associated with a reversible increase in serum creatinine of 10–15% which equates to a decline in estimated GFR of between 10 and 15 mL/min/1.73 m^2^. In individuals with diabetes, one quarter of whom had an eGFR less than 60 mL/min, this represents a very significant loss of function. This is not renal damage and is fully reversible event after 3-4 years of therapy, as observed in the FIELD trial [75]. However, it is functional renal impairment nonetheless. It has been argued that is one of the mechanisms by which fibrates might protect the kidney from overwork. However, the precise mechanism of action remains to be established. It has been suggested that PPAR*α* agonists may inhibit prostaglandin production, a key regulator of renal blood flow and diabetic renal pathology, may also be responsible [76].

## 4. The Renoprotective Effects of PPAR*γ* Agonists

### 4.1. Experimental Studies

A number of studies have suggested that PPAR*γ* agonists have direct renoprotective actions in experimental diabetes [25–31]. For example, we have shown that treatment with the PPAR*γ* agonist, rosiglitazone (20 mg/kg/day), attenuated glomerulosclerosis, tubulointerstitial expansion, and collagen IV deposition following the induction of streptozotocin diabetes (Figure 1) [25]. The increase in albuminuria and the decline in kidney function associated with diabetes in this model were attenuated in the absence of changes in glucose, insulin, or lipid levels or a reduction in blood pressure, suggesting a direct mechanism of action (see below). Similar renoprotective actions on diabetic kidney disease in other rodent models have also been observed, including in *db*/*db* mice, obese Zucker rats, and OLETF (Otsuka Long-Evans Tokushima Fatty) rats [26–31]. In other models of advanced kidney disease, such as in 5/6 nephrectomized rats, PPAR agonists are able to attenuate proteinuria and glomerulosclerosis, independent of glycemic control [77]. Even in the normal aging process, the PPAR*γ* agonist, pioglitazone can protect against normal-age-associated renal injury by reducing proteinuria, sclerosis and improving GFR [78].

### 4.2. Clinical Studies

A large number of clinical trials have demonstrated that TZDs can also reduce albuminuria patients with type 2 diabetes. A recent meta-analysis including 15 of these randomised controlled studies (5 with rosiglitazone and 10 with pioglitazone) suggested that TZD treatment, was associated with a significant fall in urinary albumin excretion [79]. The magnitude of this effect is again similar to that achieved with PPAR*α* agonists, approximately 10–20%, and appeared to be independent of improved glycaemic control. However, treatment with the PPAR*γ* agonist, pioglitazone was associated with greater decline in estimated GFR than with placebo [80].

Further evidence for a role of PPAR in diabetic kidney disease also comes from polymorphism studies in patients with diabetes and nephropathy. For example, the Pro12Ala polymorphism has been linked to a higher incidence of nephropathy in patients with type 2 diabetes [81, 82]. The same polymorphism is associated with progressive renal decline and higher rates of ESRD and all-cause mortality in patients with type 1 diabetes [83]. However, the same polymorphisms appear to influence metabolic control, meaning that a direct link cannot be assumed.

### 4.3. Potential Mechanisms of Action

PPAR*γ* activation results in increased sensitivity to the metabolic actions of insulin, partly by reversing lipotoxicity-induced insulin resistance. In addition, TZDs have been shown to “rejuvenate” pancreatic *β*-cells, reducing *β*-cell apoptosis, and increasing *β*-cell proliferation, thus maintaining *β*-cell mass and function [84]. Resistance to the actions of insulin is strongly associated with renal complications of diabetes. By reducing insulin resistance in the kidney and improving glycaemic control, PPAR*γ* activation may indirectly improve renal function. In addition, there is some evidence that insulin resistance plays a more direct role in the pathogenesis of diabetic renal disease. In particular, pathway-selective insulin resistance appears to be associated with activation of pathogenic pathways due to hyperinsulinism [85]. Indeed, recent studies show that mice with specific deletion of the insulin receptor only from their podocytes develop significant albuminuria together with histological features that recapitulate diabetic nephropathy, but in a normoglycemic environment [70]. In experimental studies performed in a rat model of type 2 diabetes, proteinuria and TGF-*β* expression in the kidney are partly attenuated by pioglitazone, without affecting glycemic control [86].

PPAR*γ* agonists also appear to have direct effects on a number of critical pathways that are implicated in the development and progression of diabetic kidney disease, over and above action on metabolic control. None of these actions occurs in isolation, but they appear synergistically intertwined, consistent with the key role of the PPAR*γ* signalling pathway as a metabolic and cellular regulator.

#### 4.3.1. PPAR*γ* and Hypertension-PPAR*γ*

Agonists may also directly impact on blood pressure [87–89], one of the key drivers of renal injury in the diabetic kidney. Despite this, experimental and clinical studies have demonstrated that PPAR*γ* activation by TZDs commonly reduces blood pressure and prevents the development of hypertension [90, 91]. In addition, the Pro12Ala polymorphism and mutations in the PPAR**γ** gene are associated with hypertension in humans [92, 93]. It has been postulated that this antihypertensive effect may be due to increase in endothelial nitric oxide biosynthesis, inhibition of the renin-angiotensin system, and/or reduced vascular inflammation [88, 90]. However, the exact mechanism remains to be established.

#### 4.3.2. PPAR*γ* and Oxidative Stress

We have previously shown that NADPH-dependent superoxide production is increased in the vasculature of diabetic mice and that this may be attenuated following treatment with the PPAR*γ* agonist, rosiglitazone [94, 95]. This is associated with reduced markers of oxidative stress, such as lipid peroxidation products [96], and improved levels of antioxidants such as glutathione reductase, glutathione, and protein carbonyl groups. PPAR*γ* agonist rosiglitazone or PPAR*γ* overexpression is also able to protect against podocyte injury inducted by aldosterone [71], potentially reflecting their role in mitochondrial biogenesis and oxidative metabolism [97]. PPAR*γ* activation is also able to attenuate AGE-induced ROS generation and decrease RAGE expression [98].

#### 4.3.3. PPAR*γ* and Renal Hypertrophy

As noted above, diabetes is characterized by maladaptive renal hypertrophy. We have previously shown that treatment of diabetic mice with the PPAR*γ* agonists, rosiglitazone, is able to reduce renal hypertrophy associated with STZ diabetes [25] and uninephrectomized db/db mice [99]. It has been suggested that activation of PPAR*γ* is able to directly modify cell cycle signaling [100]. Certainly, pioglitazone ameliorates downregulating the induction of p27 in glomerular cells and reversing high glucose-induced cell hypertrophy in OLETF rats [96]. Other effects on growth factors and indirect actions via improved glycaemic control probably also contribute to reduce renal hypertrophy observed with *PPAR*γ* agonists.*

#### 4.3.4. PPAR*γ* and Inflammation

Inflammation also plays a significant role in the development and progression of diabetic kidney disease. Treatment with TZDs is able to suppress circulating levels of cytokines in patients with diabetes [60, 61]. It has been suggested that TZDs ameliorate renal injury in diabetic rats partly through inhibition of ICAM-1 expression, NF-*κ*B activation, and macrophage infiltration in the kidney [101].

#### 4.3.5. PPAR*γ* and Podocyte Biology

The glomerular podocyte plays a key role in the development and progression of albuminuria and glomerulosclerosis associated with diabetes [66–69]. Such data place podocytes, and more particularly the dysregulation of their growth and differentiation, at the very centre of the pathogenesis of nephropathy. Podocytes clearly express PPAR; but more importantly podocyte injury may be attenuated following treatment with PPAR agonists, both *in vitro* [71] and in vivo [27, 72–74]. For example, PPAR*α* agonists are also able to increase gene expression of the key slit-pore protein, nephrin, in diabetic nephropathy, potentially contributing to their antiproteinuric actions [27]. PPAR*γ* agonists also affect nephrin gene transcription [74]. Indeed, in cultured podocytes, TZDs are able to directly reduce apoptosis and injury and improve podocyte differentiation [71]. Similarly in an immune model of progressive nephropathy, passive Heymann nephritis, the PPAR*γ* agonist, pioglitazone, had an antiproteinuric effect, possibly via transcriptional regulation of nephrin [74].

#### 4.3.6. PPAR*γ* and Renal Fibrogensis

It is thought that PPAR activation influences many compenents of fibrogenesis, including the synthesis of matrix proteins, the expression of fibrogenic growth factors like TGF-*β* and CTGF, as well as metalloprotease activity. Certainly, treatment with PPAR*γ* agonists is able to reduce glomeruloscleoriss and tubulointerstitial fibrosis in diabetic mice (Figure 1) [25]. In mesangial cells exposed to high glucose PPAR*γ* prevents the upregulation of collagen IV [102].

#### 4.3.7. PPAR*γ* and Podocyte Biology and Adiponectin

Adiponectin is widely considered to be a renoprotective adipokine. Certainly, adiponectin-deficient mice exhibit increased albuminuria and podocyte foot process effacement, which can be rescued following exogenous administration of adiponectin [103]. Adiponectin is a direct target gene of PPAR*γ*. Treatment of rosiglitazone or pioglitazone markedly increases the adiponectin levels, alongside reductions in proteinuria [104].

### 4.4. Other Renal Actions of PPAR*γ* Agonists

Treatment with PPAR*γ* agonists is limited by several common adverse effects, including substantial weight gain and fluid retention. This is thought to be the result of upregulation of the epithelial sodium channel in the kidney [105], promoting fluid retention. In addition, activation of PPAR*γ* results in activation of the sympathetic nervous system, increased endothelial permeability [106], and increased renin expression.

## 5. The Renoprotective Effects of PPAR*δ* Agonists

### 5.1. Experimental Studies

Although PPAR*δ* is ubiquitously expressed in all nephron segments, its role in the kidney remains to be fully established. The potential actions of PPAR*δ* agonists in the diabetic kidney have only been recently explored. In streptozotocin-induced diabetes, the expression of PPAR*δ* is increased in the kidney [23], associated with the development and progression of renal damage. By contrast, the selective PPAR*δ* agonist GW0742 reduces albuminuria, glomerular mesangial expansion, renal inflammation, and collagen accumulation, without significantly affecting blood glucose levels [107, 108].

### 5.2. Clinical Studies

Some studies have suggested that there is an association between the metabolic phenotype and polymorphisms of the PPAR*δ* gene [109]. In humans, PPAR*δ* agonists decrease plasma triglycerides and increase high-density lipoprotein (HDL) cholesterol [110]. In theory, treatment of dysliipidaemia would be expected to also have renoprotective benefits [38–41]. However, as yet no studies have been published to test this hypothesis.

### 5.3. Potential Mechanisms of Action

As noted above, dyslipidaemia is a major reversible risk factor for diabetic CKD [38–41]. The reversal of dyslpidaemia following treatment with PPAR*α* may be partly responsible for the renoprotective actions of these agents. Activation of the PPAR*δ* receptor is thought to alter the circulating lipid profile by enhancement of fat oxidation in skeletal muscle. PPAR*δ* knockout mice are also insulin resistant/glucose intolerant, although this may reflect lipotoxicity rather than a direct effect on insulin sensitivity. PPAR*δ* agonists may also have direct effects on a number of critical pathways that are implicated in the development and progression of diabetic kidney disease, over and above action on metabolic control.

#### 5.3.1. PPAR*δ* and Hypoxia

Some data suggest that PPAR*δ* is involved in the regulation of apoptosis in response to renal injury [111]. Deficiency of PPAR*δ* increases susceptibility to ischemic injury in the kidney, while selective agonists of PPAR*δ* protect against ischemic acute renal failure [111]. Certainly, hypoxia is known to be an important factor in the pathogenesis of diabetic kidney disease [112], implying that this effect may be important in the diabetic kidney. In addition, the expression of PPAR*δ* is increased in the diabetic kidney [107].

#### 5.3.2. PPAR*δ* and Inflammation

Activation of the PPAR*δ* receptor appears to have anti-inflammatory actions in the kidney [113]. It is known that unliganded PPAR*δ* binds to the anti-inflammatory transcriptional repressor Bcl-6, inhibiting its suppressive actions on inflammation [114]. Certainly, treatment with the PPAR*δ* agonist GW0742 reduces macrophage infiltration in the diabetic kidney [107]. Whether this is a direct effect on the kidney or on the macrophages recruited by renal injury remains to be established. Treatment of macrophages with GW0742 reverses glucose-induced induction of pro-inflammatory mediators like macrophage chemotaxis factor-1 (MCP-1). However, more recent studies have suggested that PPAR*δ* is not essential for the anti-inflammatory effect of some of the so-called selective PPAR*δ* receptor ligands, suggesting that other pathways may also be involved [113].

#### 5.3.3. PPAR*δ* and AGE/RAGE

The AGE/RAGE pathways has been strongly implicated in the pathogenesis of diabetic kidney disease. Activation of the PPAR*δ* receptor in diabetic mice is reduced following treatment with the PPAR*δ* agonist, L-165041 [108]. Certainly, L-165041 reduces AGE-mediated apoptosis and inflammation *in vitro* [115], partly by this pathway.

## 6. Conclusions

PPAR agonists have clear and reproducible renoprotective effects, impacting on a range of pathways implicated with the development and progression of diabetic kidney disease, including oxidative stress. For the TZDs, the cardiovascular toxicity, fluid retention, and risk of bladder cancer have now overshadowed these important renoprotective actions. However, the new recognition of fibrates as agents that prevent microvascular complications of diabetes will hopefully leads to renewed interest in the role of PPARs in the kidney.

The actions of the glitazars, highly potent combined PPAR *α*/*γ* agonists is also salutary. Although many times more effective activators of PPAR receptors, these agents appeared no more effective than currently available agonists, while having a poorer side effect profile. It may be that PPAR agonists also have activities through PPAR-independent pathways, possibly via the transrepression of pathogenic genes, such as activator protein-1 (AP-1) and nuclear factor-*κ*B (NF-*κ*B) [6]. Such activity may only loosely correlate with activation of the PPAR receptor [7]. Greater understanding of the exact mechanism of this pleiotropism will open the way for new therapeutics for the prevention of diabetic kidney disease.
